# Combined application of alginate dressing and human granulocyte-macrophage colony stimulating factor promotes healing in refractory chronic skin ulcers

**DOI:** 10.3892/etm.2014.1652

**Published:** 2014-03-31

**Authors:** GUOBAO HUANG, TANGQING SUN, LEI ZHANG, QIUHE WU, KEYAN ZHANG, QINGFEN TIAN, RAN HUO

**Affiliations:** 1 Department of Burn and Plastic Surgery, Provincial Hospital Affiliated to Shandong University, Jinan, Shandong 250021, P.R. China; 2 Department of Burn and Plastic Surgery, Jinan Central Hospital Affiliated to Shandong University, Jinan, Shandong 250013, P.R. China

**Keywords:** alginate, GM-CSF, refractory skin ulcer, diabetic foot

## Abstract

The aim of the present study was to evaluate the clinical therapeutic effect of the combined application of alginate and recombinant human granulocyte-macrophage colony-stimulating factor (rhGM-CSF) on the healing of refractory chronic skin ulcers. A single center, three arm, randomized study was performed at Jinan Central Hospital (Jinan, Shandong, China). A total of 60 patients with refractory chronic skin ulcers, which persisted for >1 month, were enrolled and randomly assigned into one of the following three groups: alginate dressing/rhGM-CSF group (group A), rhGM-CSF only group (group B) and conventional (vaseline dressing) group (group C). The wound area rate was measured, granulation and color were observed and pain was evaluated. The data were summarized and statistical analysis was performed. The results demonstrated that group A exhibited a significantly faster wound healing rate and lower pain score compared with the other groups (P<0.01). In conclusion, the combined application of alginate dressing and rhGM-CSF for the treatment of refractory chronic skin ulcers demonstrated significant advantages. It promoted the growth of granulation tissue, accelerated re-epithelialization and also effectively reduced wound pain, and thus improved the quality of life for the patient. This suggests that the combined application of alginate and rhGM-CSF may be an effective therapeutic strategy for the clinical treatment of refractory chronic skin ulcers.

## Introduction

The treatment of refractory chronic skin ulcers is challenging due to the different causes and characteristics of the wounds. Significant efforts have been made in the development of a variety of treatment materials and processes where multiple factors determine the drug used, including familiarity with the products, characteristics of the patient and the cost (1,2). Alginate is a natural anionic polymer typically extracted from seaweed and has been investigated and used for numerous biomedical applications. The advantages of alginate include biocompatibility, low toxicity, relatively low cost and mild gelation when a divalent cation such as Ca^2+^ is added (3). The structural similarity of alginate to extracellular matrices of living tissues allows wide applications in wound healing, delivery of bioactive agents (including, small chemical drugs and proteins) and cell transplantation. It has previously been verified that alginate application alone does not facilitate wound healing compared with other traditional methods (4), however, alginate has been used in combination with other drugs or protein factors for the treatment of wound healing (5–11).

Human granulocyte-macrophage colony stimulating factor (hGM-CSF) is a multifunctional growth factor and a mitogenic agent that has been demonstrated to be involved in a number of essential processes of wound healing (12,13). Recombinant hGM-CSF (rhGM-CSF) has been demonstrated to promote the healing of infected burn wounds, as well as prevent infections by modulating immune activity and improving immune competence (14). The first use of locally-delivered rhGM-CSF in chronic wounds was reported in 1994 (15). Clinical studies and case reports have demonstrated that rhGM-CSF has a positive effect on chronic wounds with varying etiologies, including chronic venous ulcers, pressure ulcers, erythropathy-associated ulcers, neutrophil dysfunction-associated chronic ulcers, immunodeficiency-associated ulcers, leprosy ulcers and refractory wounds in patients with cancer (12). However, it has previously been demonstrated that rhGM-CSF does not have a significant effect on healthy wounds (16).

In order to achieve optimal results, rhGM-CSF must be continuously present in the wound at a certain concentration. In the present study, the combined effect of alginate and rhGM-CSF on the treatment of refractory chronic skin ulcers was investigated. It was hypothesized that the combination of alginate and rhGM-CSF significantly enhances wound healing compared with the treatment of rhGM-CSF alone.

## Patients and methods

### Patients

A single center, three-arm, randomized clinical study was performed. Patients with refractory chronic skin ulcers were enrolled between October 2009 and March 2012 from Jinan Central Hospital (Jinan, Shandong, China). The present study was approved by the Ethics Committee of the Jinan Central Hospital and written informed consent was obtained from every patient. Patients with bedsores, varicose ulcers and diabetic foot ulcers, which persisted for >1 month following conventional and anti-inflammatory treatments, and with a wound area >10 cm^2^, were selected for the present study. Patients with the following criteria were excluded from the present study: i) fasting plasma glucose levels >10.0 mmol/l, even following strict control; ii) patients with severe cardiac dysfunction (≥level III), severe renal dysfunction (≥level II), as well as severe diseases, including cancer, tuberculosis, chronic atrophic gastritis and systemic lupus erythematosus.

Patients were randomly assigned to one of the following three treatment groups: group A, alginate dressing plus rhGM-CSF; group B, rhGM-CSF only; and group C, conventional treatment group using a vaseline gauze.

### Treatment

Following cleaning the wounds with saline and drying them with sterile cotton, rhGM-CSF paste (Changchun Jinsai Pharmaceutical Co., Ltd., Changchun, Jilin, China) containing 100 μg rhGM-CSF/10 g, covered by alginate dressing (Smith & Nephew, London, UK) was applied over the wound area for patients in group A. In group B, rhGM-CSF paste covered by a vaseline gauze was applied to the wounds, whilst in group C only a vaseline gauze was applied. The primary dressings were covered with cotton gauzes and bandages and the dressings were changed daily for the first 7 days and every other day thereafter.

### Wound evaluation

Visual observations were made on the wound color and the growth of granulation. The physicians who performed the evaluation were blinded to the treatment of the patients. The evaluation criteria were determined and scored on a four-point scale: 4 points, rosy colored wound surface and well-developed granulation tissue; 3 points, pink wound surface with moderate granulation tissue growth; 2 points, dark red wound surface with light granulation tissue growth; and 1 point, pale wound surface without granulation. The average scores for all the individuals in each group were calculated and expressed as the group healing score (GHS).

The wound area was measured and recorded each time the dressing was changed. Healing was assessed as the percentage area that was healed [(pre-treatment area - post-treatment area)/pre-treatment area]. The ease with which the dressing was removed was also assessed, whilst the patient scored the comfort of the procedure. The dressing changes and treatment assessments were performed by unbiased nurses.

Pain was assessed using the visual analogue scale (VAS) method. A 10 cm linear score was used, where 0 indicated painless and 10 indicated intolerable pain. Patients marked on the line based on their pain intensity.

### Statistical analysis

The data were analyzed using SPSS statistical software (PASW statistics version 18.0; IBM, Armonk, NY, USA). Differences in the outcome variables were analyzed on an intention-to-treat basis. Differences in wound healing time between the groups were examined using the Chi-square test. P<0.05 was considered to indicate a statistically significant difference.

## Results

### Patient demography data and general disease situation

A total of 60 patients were enrolled in the present study, including 35 males and 25 females, aged between 20 and 75 years with an average age of 50.6. In total, 25 patients had pressure sores, 15 had varicose ulcers and 20 had diabetic foot ulcers. The ulcer area ranged between 11 and 35 cm^2^, with an average size of 17.2±8.0 cm^2^. The duration of the wound was 1–3.5 months, with an average time of 1.8±2.1 months prior to the start of the study. Patients were randomly assigned into one of the three treatment groups shown in Table I. Statistical analysis demonstrated no significant difference between the patients in each treatment group.

### Visual observation

**Figure 1:**
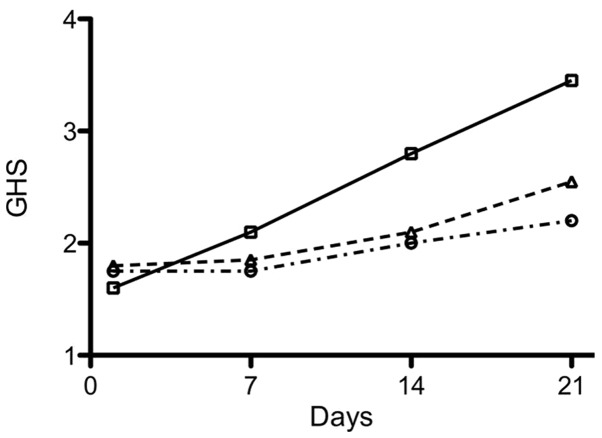
Quantified evaluation of wound healing using the GHS based on visual observations of patients in groups A (solid line), B (dashed line) and C (dashed dot line). GHS was calculated as the average score of a group at an evaluation time point. Statistical analysis demonstrated significant differences among the three groups (P<0.001). GHS, group healing score.

Pre-treatment assessment was performed on the first day and was used as baseline for each patient. Observations were routinely performed each time the dressing was changed by physicians who were blinded to the treatment of the patients, and the data are summarized in Table II. An increase in granulation tissue growth and color changes were observed at multiple time points in group A (alginate + rhGM-CSF) and B (rhGM-CSF) compared with group C (vaseline only). The GHS for each group was calculated and the results are shown in Fig. 1. The differences between the groups were found to be statistically significant (P<0.05; Table II). The ease of changing the dressing and the comfort of the patient were assessed and no significant differences were identified among the three groups (data not shown).

### Wound healing

**Figure 2:**
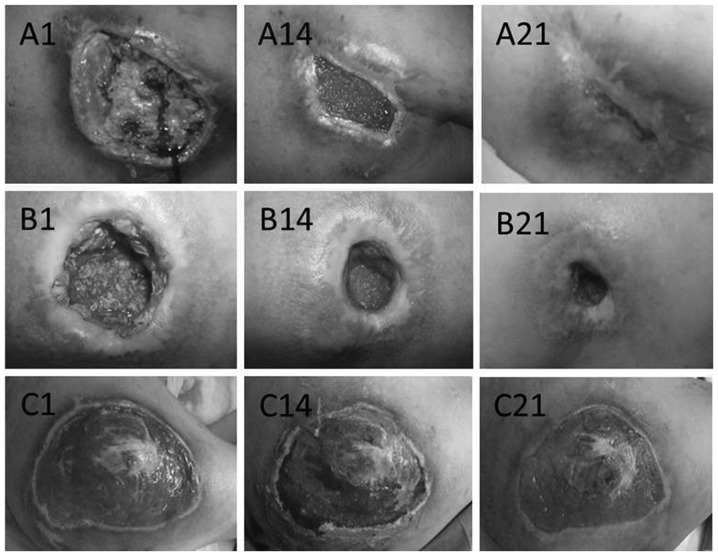
Representative images of pressure sores from patients in the three treatment groups at different time points. A, B and C indicate the treatment group that each image represents and the number indicates the number of days of treatment. The images demonstrate wound surface color, granulation and excretion, however, the wound surface area is not demonstrated since the magnitude of each image may be different.

**Figure 3:**
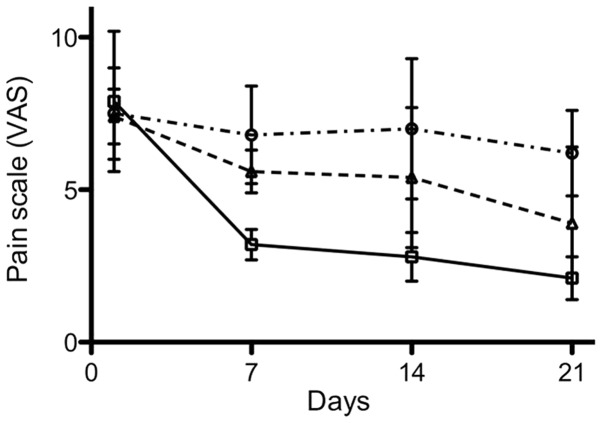
Wound healing rate, calculated by measuring the wound area at designated time points, is shown for patients in groups A (solid line), B (dashed line) and C (dashed dot line). The wound area was measured each time the dressing was changed. The length of the longest diameter and the vertical diameter were obtained. Statistical analysis demonstrated significant differences among the three groups (P<0.001).

Representative cases from each group are shown in Fig. 2 where differences between the groups are illustrated. The wound healing rate was calculated and the data demonstrated significant differences among the three groups at all assessment time points. Following 3 weeks of treatment, group A demonstrated the highest healing rate of 56%, whilst group C and B had a healing rate of 21 and 34%, respectively (Fig. 3). Following 7 days of treatment, group A exhibited a rapid growth of fresh epithelium towards the wound center and clear wound contraction and re-epithelialization. Group B showed moderate wound contraction compared with group A, however, this was significantly greater compared with group C. The differences were statistically significant among the three groups (P<0.05; Fig. 3).

### Pain evaluation

**Figure 4:**
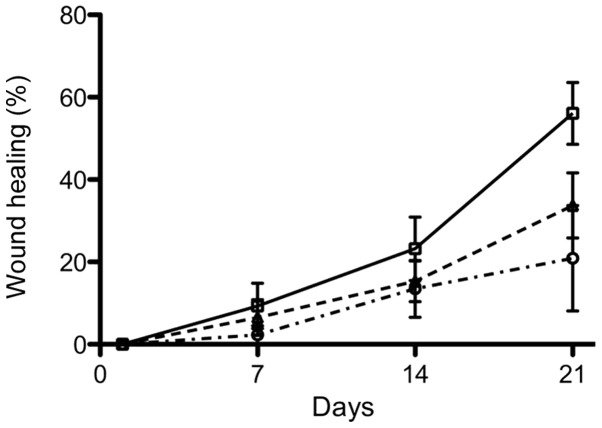
Pain evaluation was performed using the VAS method. Patients in groups A (solid line), B (dashed line) and C (dashed dot line) were asked to assess their pain on a scale each time the dressing was changed. The data were plotted at different time points. Statistical analysis demonstrated significant differences among the three groups (P<0.001). VAS, visual analogue scale.

Wound pain was assessed using the VAS method on a 10 cm scale. Group A showed significantly reduced pain compared with the other groups (Fig. 4), and the differences among the three groups were statistically significant (P<0.05).

## Discussion

In the present study, the treatment of refractory chronic skin ulcers using a combination therapy of rhGM-CSF with sodium alginate was investigated. rhGM-CSF has been previously demonstrated to be a mitotic-promoting reagent capable of promoting wound healing. hGM-CSF was first used by da Costa *et al* (15) to treat patients with chronic refractory wounds on lower extremities and promising results were observed. In a randomized, double-blind, placebo-controlled trial on ulcers caused by varicose veins, it was found that hGM-CSF treatment was significantly improved compared with the placebo control treatment, and no adverse reactions were observed. rhGM-CSF has also been demonstrated to be effective for the treatment of refractory chronic wounds accompanied with hereditary leukocyte dysfunction (17), post-surgical wounds (18) and hereditary neutrophil dysfunction (19), as well as for the treatment of pyoderma gangrenosum (16,20).

Alginate is used for the treatment of wounds as it absorbs the exudate from the wound and exchanges Na^+^/Ca^2+^ ions with the exudate, forming a gel over the wound (21). Through the ion exchange, insoluble calcium alginate is converted into soluble sodium alginate. Soluble sodium alginate is capable of absorbing a 20-fold amount of its own weight of exudate (gauze absorbs 5–7 times) and forms a soft, moist, semi-solid gel-like substance, in order to keep the wound isolated from infection or further harm. It is known that wound healing occurs more rapidly when a gel is formed at the wound surface and dehydration is prevented (22). Besides maintaining a moist environment, alginate dressings possess other characteristics that are beneficial for wound healing, including good permeability, a lack of toxicity, stimulation and antigenicity, the ability to prevent bleeding and promote clotting as well as the ability to reduce water, salt and nutrient loss from the wound surface and inhibit the growth of bacteria (23). The alginate gel also accelerates microvessel hyperplasia and promotes granulation tissue formation and rapid epithelialization (24). Therefore, alginate has been widely used in various medical conditions (25,26), even though alginate alone does not directly enhance the wound healing process (4). The data from the present study suggested that the combined use of alginate and rhGM-CSF reduced the wound healing time and significantly decreased the discomfort of the healing process. This suggests that alginate may be able to create an improved environment so that rhGM-CSF is able to reach its maximum effect.

Several previous studies have been performed using rhGM-CSF with intradermal or subcutaneous injection around the wound (20,27–31). However, there are clear disadvantages caused by the injection, including pain and uneven distribution of rhGM-CSF in the wound area, which has prevented it from being widely used. Topical application of rhGM-CSF is likely to cause loss of rhGM-CSF due to dilution with the wound secretions. Therefore, the soft gel formed by alginate is the ideal material to prevent this from happening. In addition, the gel enables the continuous presence of rhGM-CSF on the wound surface for a longer length of time, which accelerates healing and reduces the quantity of rhGM-CSF applied to the wound surface. Therefore, the combination of alginate dressings with rhGM-CSF is theoretically superior to treatment with rhGM-CSF alone, and the results from the present study support this theory.

In the present study, patients with pressure sores, varicose ulcers and diabetic foot ulcers were enrolled, and each patient was randomly assigned to each group. However, statistical analysis to assess the differences based on the type of wound was not performed due to the limited number of cases. The results from the present study would have been more conclusive if patients with only one medical condition were selected. In addition, due to certain technical reasons the wound depth was not measured and instead the wound area was used as the sole standard for wound healing evaluation. Thus, further studies are required to validate the results from the present study.

In conclusion, the present study demonstrated that alginate-rhGM-CSF dressing for refractory chronic skin ulcers promoted the growth of granulation tissue, accelerated re-epithelialization, whilst also effectively reducing wound pain. These results suggest that the combination of the two may be used for the routine treatment of refractory chronic skin ulcers.

## Figures and Tables

**Table I:** Demographic data and medical history of the patients.

Group	Number of patients	Males/females	Age (years)	Wound duration (months)	Pressure sores	Varicose ulcers	Diabetic foot	Ulcer area (cm^2^)
A	20	12/8	55.2±20.4	2.1±2.0	8	4	8	17.8±11.5
B	20	13/7	49.9±10.5	1.9±2.4	7	8	5	16.4±7.6
C	20	10/10	50.6±10.0	1.7±1.7	10	3	7	17.0±7.5

**Table II:** Assessment of wound color and granulation at selected time points.

	Group A				Group B				Group C			
			
Time (days)	1	2	3	4	1	2	3	4	1	2	3	4
1	11	6	3	0	9	9	2	0	8	9	3	0
7	7	6	5	2	7	10	2	1	7	11	2	0
14	2	5	8	5	5	9	5	1	5	10	5	0
21*	0	2	11	8	3	6	8	3	5	8	5	2

* P<0.001 comparing Group A with Group B and C at 21 days.
